# Understanding injecting drug use in Afghanistan: A scoping review

**DOI:** 10.1186/s13011-022-00491-1

**Published:** 2022-09-19

**Authors:** Frishta Nafeh, Sufiat Fusigboye, Bundit Sornpaisarn

**Affiliations:** 1 Department of Public Health Sciences, Queen’s University, 62 Fifth Field Company Lane, Carruthers Hall, Kingston, Ontario K7L 3N6 Canada; 2 Centre for Addiction and Mental Health, Institute for Mental Health Policy Research, Toronto, ON Canada; 3 Dalla Lana School of Public Health, University of Toronto, Toronto, ON Canada; 4 Faculty of Public Health, Mahidol University, Bangkok, Thailand

**Keywords:** People who inject drugs, Injecting drug use, Harm reduction, Addiction, Global health, South Asia, Afghanistan

## Abstract

**Background:**

Several reports have described a growing prevalence of illicit drug use in Afghanistan, with recognition of a recent shift from traditional modes of consumption involving inhalation and oral ingestion to injecting drug use.

**Objective:**

Conduct a comprehensive review of existing literature to map the injecting drug use situation in Afghanistan. The review intends to describe risk factors and impacts of injecting drug use, drug use characteristics and risk behaviours among people who inject drugs (PWID), and access to harm reduction and treatment.

**Methods:**

We searched Embase, Global Health, Medline, PsycINFO, Web of Science, and grey literature to identify English language publications up to March 26^th^, 2022. Studies were eligible for inclusion if they explicitly targeted PWID or injecting drug use in Afghanistan and provided information relevant to the review questions. Two reviewers independently screened titles and abstracts for inclusion and extracted information based on the review objectives.

**Results:**

A total of 25 articles were identified representing 15 studies (11 quantitative, 2 qualitative, 2 mixed methods). All but one studies were cross-sectional. In majority of the studies, over 95% of the participants were male and most were conducted over a decade ago, in urban settings, mainly Kabul. Only one study examined risk factors of injecting drug use. Eleven studies described drug use characteristics and 9 reported risk behaviours among PWID. Health and social burden of injecting drug use were reported by 8 and 5 studies, respectively. Nine studies described access to harm reduction and treatment. Afghan PWID had high levels of injecting and sexual risk behaviours compared to global estimates. They reported high prevalence of incarceration and displacement. Access to harm reduction and treatment was very limited. This scoping review revealed important knowledge gaps including a gender gap in research with serious implications for drug policy and substance use care.

**Conclusions:**

Development of a national public health-oriented drug policy and substance use care programme is warranted along with efforts to develop health research capacity to address the need for epidemiological data. The current humanitarian crisis necessitates continued access to evidence-based harm reduction and treatment in Afghanistan.

**Supplementary Information:**

The online version contains supplementary material available at 10.1186/s13011-022-00491-1.

## Introduction

Since the mid 1990s, Afghanistan has come to be known as the world’s largest producer of opium with an estimated 85% share of the total global supply in 2020 [1, 2]. Opium poppy cultivation has been steadily increasing in recent decades, with an average annual increase of 4,000 hectares since the start of systematic monitoring in 1994 [3]. Furthermore, preliminary evidence indicates emergence of a growing illicit methamphetamine market in Afghanistan, after manufacturing rose in 2016 [4]. For instance, data on drug seizures showed an increasing percentage of methamphetamine seized in neighbouring countries originated in Afghanistan between the periods 2014–2018 and 2019–2021 [5, 6]. Given the burgeoning illicit drug economy in Afghanistan with opiates making up to 14% of the national gross domestic product (GDP) in 2021 [5], it is inevitable that drug use is a considerable public health problem in Afghanistan. The availability of cheap illicit drugs [5] coupled with rampant poverty [7] and decades of war – contributing to large-scale population displacement [8] and widespread psychological distress [9, 10] – puts many Afghans at risk of problematic drug use and subsequent health and social consequences [11, 12].

The extant literature – albeit limited – points to a rapidly growing drug use problem of potentially epidemic proportions. According to Afghanistan's first ever population-based drug use survey conducted in 2005, 3.8% of the population reported illicit drug use with the most common drugs being cannabis followed by opiates [13]. In 2009, a follow-up survey reported a drug use prevalence of 8% among adults 15–64 years of age, along with a 53% increase in the number of regular opium users and an increase of 140% in the number of regular heroin users since 2005 [14]. In 2015, a new survey (Afghanistan National Drug Use Survey) that used confirmed biological measures found a national drug use prevalence of 12.8% among those 15 years and older [15] compared to a global rate of 5.3% among adults of the same age in the same year [16]. According to Afghanistan National Drug Use Survey in 2015, opioids became the most used illicit drugs in Afghanistan with a prevalence of 8.5%, exceeding the opioids prevalence in North America (4.42%) [15, 16].

Among illicit drugs, opioids are the most harmful, causing the highest burden of morbidity and mortality attributable to drug use disorders [17]. Between 2016–2019, over 70% of disability adjusted life years (DALYs) attributable to drug use disorders were due to opioids alone [2, 17]. Opioids are the most injected drugs with a global prevalence of 83% among people who inject drugs (PWID) [18]. Route of drug administration has important implications for health with injection being the riskiest route. Compared to non-injection drug users, PWID have elevated risk of drug dependence [19, 20], frequent overdoses [21, 22], and all-cause mortality [23]. While inhalation and oral ingestion was historically the most common routes of drug administration in Afghanistan, emerging evidence suggests that displacement and migration to and return from neighbouring countries of Pakistan and Iran have contributed to a growing trend in injecting among drug users in Afghanistan [24].

The high prevalence of illicit opioid use in a nation with one of the world’s worst public health and socioeconomic indicators [25, 26] and punitive laws often dictated by religion around substance use [27, 28] may lead to devasting health and social outcomes for people who use drugs in general and PWID in particular. Afghanistan has high rates of communicable diseases and is endemic for malaria, viral hepatitis, and emerging concentrated human immunodeficiency virus (HIV) among drug users [29, 30]. Health indicators are further challenged by a severe lack of social and health infrastructures due to decades of war and poverty [31]. Therefore, it is critical to understand the nature and extent of injecting drug use (IDU) in Afghanistan and identify priority areas for future research.

Several global literature reviews have described the epidemiology of IDU, some of which have provided numerical information for Afghanistan; however, these lack context and interpretation [32–37]. Therefore, the present review was undertaken to identify and map the existing literature on IDU in Afghanistan by adopting a scoping review design, which is more suitable for broad objectives that aim to identify, describe, and/or map the current body of evidence on a topic [38, 39]. Consistent with *the Joanna Briggs Institute (JBI) Manual for Evidence Synthesis*, the Population, Concept, and Context Framework was used to guide the review questions [40]. The present scoping review intends to answer the following review questions:

1. What are the risk factors of IDU in Afghanistan?
2. What are the common drug use characteristics and risk behaviours among PWID in Afghanistan?
3. What is the health and social burden of IDU among PWID in Afghanistan (e.g., comorbidity, overdose, mortality, incarceration, stigma, etc.)?
4. What is the evidence on access to harm reduction and treatment service among PWID in Afghanistan?

## Methods

This scoping review followed a framework proposed by the JBI [40], which builds on previous guidance developed by Arksey and O-Malley (2005) [39] and Levac et al. (2010) [41]. In addition to the JBI framework, the *“Preferred Reporting Items for Systematic Reviews and Meta-Analyses (PRISMA) extension for Scoping Reviews”* (PRISMA-ScR) [42] was also used to guide the reporting of this review (see Supplementary Appendix B). A scoping review protocol was developed a priori, to ensure the review methods were transparent and reproduceable. The protocol is available upon request from the corresponding author.

### Literature review and data sources

**Fig. 1:**
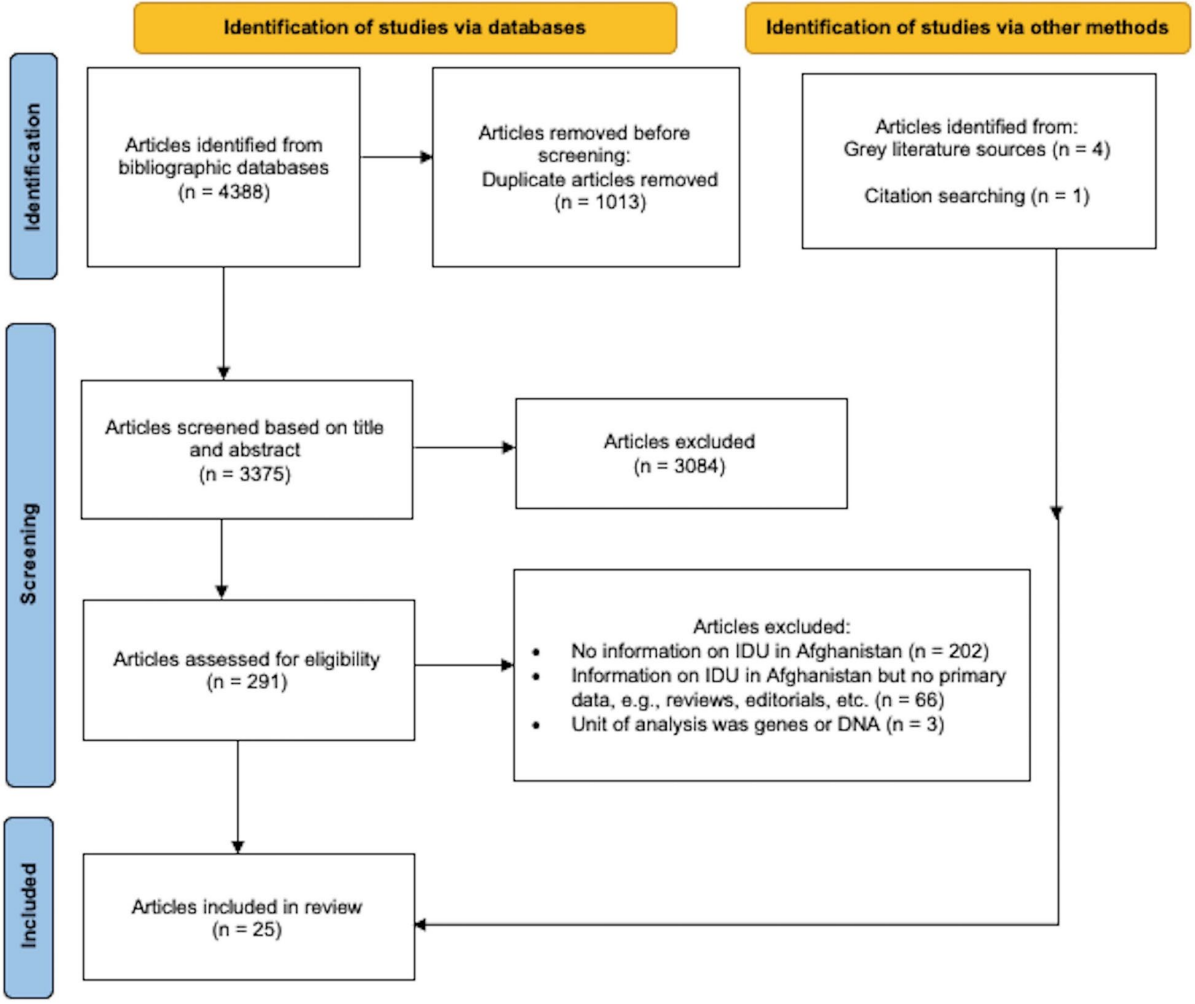
Preferred Reporting Items for Systematic Reviews and Meta-Analyses (PRISMA) Flow Diagram of Articles Selection Process. The bibliographic database searches yield 3375 unique articles. Based on title and abstract screening, 3084 articles were excluded and 291 underwent full-text screening. After full-text screening based on the inclusion criteria, 20 articles were included in the scoping review. Grey literature and citation searching of included studies yielded another 5 articles. PRISMA Flow diagram adapted from Page, M.J., McKenzie, J.E., Bossuyt, P.M., Boutron, I., Hoffmann, T.C., Mulrow, C.D., et al. (2020). The PRISMA 2020 statement: An updated guideline for reporting systematic reviews. *BMJ*, 372, n71

A comprehensive search algorithm was developed and pretested to capture relevant literature. The final search algorithm included two components, one pertaining to “IDU” and the other pertaining to “Afghanistan”. See Supplementary Appendix A for additional information on the search strategies. Searches were executed on March 26^th^, 2022, using five bibliographic databases: Embase, Global Health, Medline, PsycINFO, and Web of Science. To provide a more comprehensive and timely view of available evidence [43, 44], grey literature was searched using a systematic approach consistent with technical guidelines developed by the National Drug and Alcohol Research Centre [45]. We also searched the reference lists of all the selected articles to identify additional relevant documents. See Fig. 1 for the PRISMA flow diagram.

### Inclusion and exclusion criteria

The inclusion criteria consisted of studies or reports (written in English) with primary data generated by study authors or reliable government and/or research organizations. In addition, studies or reports were eligible for review if they explicitly targeted PWID or IDU in Afghanistan and provided information relevant to the review questions. Furthermore, conference proceedings were considered for inclusion if they provided information relevant to the review questions. There was no restriction on publication date due to the dearth of published literature on drug use in Afghanistan; we searched for all available articles until March 26^th^, 2022. In terms of study design, we included both quantitative and qualitative studies. Exclusion criteria included unit of analysis not being individuals (PWID) or country (Afghanistan), aggregated information or data (e.g., regional estimates), study population being Afghans residing outside Afghanistan (refugees and immigrants), and study setting not being Afghanistan. In addition, publications about PWID that did not stem from primary research (e.g., reviews, commentary, opinions, or editorials) as well as study protocols were also excluded.

### Data extraction

Publication details from the bibliographic databases were imported into the reference management software, Endnote desktop, where duplicates were removed manually. All articles underwent a two-step screening process, whereby titles and abstracts were initially screened for full-text review if they addressed the target population ‘people in Afghanistan’ and/or ‘IDU/PWID’. In the second step, the full text of selected titles and abstracts were examined and selected for review based on the inclusion and exclusion criteria. The screening process was carried out independently by FN and SF. Discrepancies in the screening process were discussed and resolved by the review team.

A data charting form (using Excel spreadsheet) was developed by FN a priori and pilot tested. The data charting form included identification information (author(s); publication date), methodological information (study year, location, design; sampling method; sample size), main findings, limitations as well as information pertaining to the review questions. Data charting was performed independently by FN and SF. Discrepancies in the data charting process was discussed and resolved by the review team.

### Collating, summarizing, and reporting results

The results were organized as themes guided by the review questions. The thematic categories included risk factors of IDU, drug use characteristics and risk behaviours among PWID, health and social burden of IDU, and access to harm reduction and treatment services. Risk factors included any personal and/or structural determinants of IDU. Drug use characteristics included any description of drug use patterns (e.g., type of drugs used, age of drug use initiation, frequency and duration of drug use, place of drug consumption, etc.) and risk behaviours included any sexual and drug use related activity that put PWID at risk of health harms such as infectious disease transmission, overdose, and mortality. Health burden included any comorbidity and mortality among PWID, while social burden included incarceration, criminalization, financial issues, and experiences of stigma. Themes were presented based on how they emerged in the selected studies.

## Results

### Characteristics of included studies

The flow diagram of eligible studies is shown in Fig. 1. In total, 25 articles (20 peer-reviewed papers and 5 reports) met the inclusion criteria and covered 15 studies (see Table 1). The study dates ranged from 2005–2019, with majority (9 out of 15) conducted over a decade ago, prior to 2012 (Table 1). Eleven studies were quantitative, 2 were qualitative, and 2 were mixed-methods design (Table 1). All but one of the quantitative studies were cross-sectional with one observational cohort study (Table 1). One of the mixed-methods studies included in-country assessments involving key informant interviews, focus groups, and field observations, supplemented by desk reviews [46]; unit of analysis was country. The other mixed methods study utilized structured, interviewer-administered questionnaire and focus groups [47]. In most studies (*n* = 8; representing 17 publications), participants were convenience samples of PWID recruited through harm reduction services or by harm reduction and/or outreach workers known to them [48–64]. In terms of geographic representation, only one study included participants from rural areas [14]. Over half (8 out of 15) of the studies were conducted in the capital city of Afghanistan, Kabul alone (Table 1). Majority of the studies (*n* = 11) included male PWID participants only; in 2 studies, over 95% of the sample were male (Table 1). Only one study was exclusively about women who inject drugs [53]. Table 1 outlines key characteristics of the included studies.

**Table 1:** Key characteristics of the studies included in the scoping review

Study Number	Author & Publication Year	Study Year	Study Location	Study Design	Sample Size	Sample Characteristics	Thematic Area
1	Bautista et al., 2010 [48]	2005–2006	Kabul	Quantitative; Cross-sectional	459	Male, age ≥ 18, reported IDU in the last 6 months (confirmed by injection stigmata)	Prevalence of HIV, hepatitis B, hepatitis C, and syphilis
	Todd et al., 2007 [49]	2005–2006	Kabul	Quantitative; Cross-sectional	463	Male, age ≥ 18, reported IDU in the past 6 months (confirmed by injection stigmata)	Prevalence of HIV, hepatitis B, hepatitis C, and associated risk behaviours
	Todd et al., 2007 [50]	2005–2006	Kabul	Quantitative; Cross-sectional	463	Male, age ≥ 18, reported IDU in the past 6 months (confirmed by injection stigmata)	HIV knowledge and awareness and their association with residence outside Afghanistan
	Todd et al., 2008 [51]	2005–2006	Kabul	Quantitative; Cross-sectional	463	Male, age ≥ 18, reported IDU in the past 6 months (confirmed by injection stigmata)	Prevalence and correlates of needle/syringe sharing among PWID
	Todd et al., 2009 [52]	2005–2006	Kabul	Quantitative; Cross-sectional	463	Male, age ≥ 18, reported IDU in the past 6 months (confirmed by injection stigmata)	Prior utilization of harm reduction and addiction treatment services
2	Burrows et al., 2019 [46]	2017–2018	Kabul	Mixed methods (qualitative interviews, field observations & desk review)	8–10 participants per focus groups (2–3 focus groups)	Not applicable (unit of analysis is country (Afghanistan))	Access to harm reduction services/resources
	Burrows et al., 2021 [65]	2018	Kabul	Mixed methods (qualitative interviews, field observations & desk review)	8–10 participants per focus group (2–3 focus groups)	Not applicable (unit of analysis is country (Afghanistan))	Access to harm reduction services/resources
3	MENAHRA, 2013 [53]	2013	Kabul	Qualitative (in-depth interviews)	10 women who inject drugs 4 key informants (subject matter experts)	Women, age ≥ 18, reported IDU in the last 12 months	Various themes related to access to harm reduction services among women who inject drugs
4	Nasir et al., 2011 [54]	2006–2008	Herat, Jalalabad, Mazar-i-Sharif	Quantitative; Cross-sectional	615	Male, age ≥ 18, reported IDU in the last 6 months (confirmed through injection stigmata)	Prevalence and correlates of HIV, hepatitis B, and hepatitis C infection
	Nasir et al., 2011 [55]	2006–2008	Herat, Jalalabad, Mazar-i-Sharif	Quantitative; Cross-sectional	615	Male, age ≥ 18, reported IDU in the last 6 months (confirmed through injection stigmata)	Comparing PWID with and without hepatitis C virus viremia
	Sanders-Buell et al., 2010 [56]	2006–2008	Kabul, Jalalabad, Herat, Mazar-i-Sharif	Quantitative; Cross-sectional	10	Male, age ≥ 18, reported IDU in the last 6 months (confirmed through injection stigmata)	HIV genotypes among PWID and sex workers
	Sanders-Buell et al., 2013 [57]	2006–2008	Jalalabad, Herta, Mazar-i-Sharif	Quantitative; Cross-sectional	113	Male, age ≥ 18, reported IDU in the last 6 months (confirmed through injection stigmata)	Circulating hepatitis C virus (HCV) genotypes and genetic linkages among HCV positive PWID
5	Rasekh et al., 2019 [66]	2016	Kabul	Quantitative; Cross-sectional	410 PWUD of which *n* = 55 were PWID	Male, age ≥ 18, receiving treatment for drug use	Prevalence and risk factors of HIV, hepatitis B, hepatitis C infection among PWUD Risk factors of IDU among PWID
6	Rasheed et al., 2022 [67]	2018–2019	Kabul, Jalalabad, Herat, Mazar-i-Sharif, Kunduz, Faizabad, Kandahar, Zaranj	Quantitative; Cross-sectional	1378	99% (*n* = 1369) male, age 15–64, reported IDU in the past 12 months	Mapping and prevalence estimation of PWID in Afghanistan
7	Ruiesenor-Escudero et al., 2014 [68]	2009	Kabul, Herat, Mazar-i-Sharif	Quantitative; Cross-sectional	548	Male, age ≥ 18, reported IDU in the last 3 months	Prevalence of HIV, hepatitis C and other infectious disease; correlates of HIV and hepatitis C infection
8	Ruiesenor-Escudero et al., 2015 [58]	2010–2012	Kabul	Quantitative; Cross-sectional	95	Male, age ≥ 18, reported current heroin injection and enrolled in opioid substitution therapy (OST)	Evaluation of OST pilot programme (characteristics of OST participants; factors associated with programme retention)
9	Todd et al., 2009 [59]	2009	Kabul	Qualitative (focus groups and free-list interviews)	2 focus groups with PWID (*n* = 20) Free-list interviews with PWID (*n* = 61)	Male, age ≥ 18, reported IDU in the last 6 months	Various themes were explored to describe the current context of IDU and available harm reduction programmes
10	Todd et al., 2010 [60]	2005–2008	Kabul, Jalalabad, Herat, Mazar-i-Sharif	Quantitative; Cross-sectional	1078	Male, age ≥ 18, reported IDU in the last 6 months (confirmed through injection stigmata)	Prevalence and correlates of syphilis and condom use
11	Todd et al., 2011 [61]	2007–2009	Kabul	Quantitative; Observational cohort	483	Male, age ≥ 18, reporting IDU in the past 30 days	Prevalence and correlates of HIV, hepatitis B, hepatitis C, and syphilis Prevalence and corelates of harm reduction programme use
	Todd et al., 2015 [62]	2007–2009	Kabul	Quantitative; Observational cohort	483	Male, age ≥ 18, reporting IDU in the past 30 days	HIV, hepatitis C, and mortality incidence and predictors Needle and syringe programme usage
	Todd et al., 2016 [63]	2007–2009	Kabul	Quantitative; Observational cohort	386	Male, age ≥ 18, reporting IDU in the past 30 days	Factors influencing risk behaviours among PWID
12	UNDOC, 2009 [14]	2009	32 provincial capitals, 354 district centres	Quantitative; Cross-sectional	2609 PWUD of which *n* = 148 were PWID	PWUD sample: 97% (*n* = 2534) male, age ≥ 18, reporting drug use in the past 12 months PWID sample: No gender or sex composition provided, age ≥ 18, reporting lifetime IDU	Prevalence of IDU; Risk behaviours; Access to harm reduction services
13^a^	UNDOC, 2014 [47]		17 provinces	Mixed methods (interviewer-administered questionnaire and focus groups)	3163 PWUD of which *n* = 32 were PWID	PWUD sample: 75% (*n* = 2388) male, age ≥ 18, reporting drug use for ≥ 6 months PWID sample: Male, age ≥ 18, reporting injecting heroin^b^	Risk behaviour (needle/syringe sharing)
14	Vogel et al., 2012 [64]	2009	Kabul	Quantitative; Cross-sectional	30 Opiate users of which *n* = 23 were PWID	PWID sample: Male, ≥ 18, reporting lifetime IDU	Drug use characteristics
15^a^	World Bank, 2008 [69]	2006–2007	Kabul, Jalalabad, Mazar-i-Sharif	Quantitative; Cross-sectional	Not specified for the social mapping exercise 76 (survey with IDU in Jalalabad & Mazar-i-Sharif)	Male, age ≥ 18, reported active/current IDU	Mapping and prevalence estimation of PWID; drug use characteristics; risk behaviours; access to harm reduction services

*IDU* Injecting Drug Use, *PWID* People Who Inject Drugs, *PWUD* People Who Use Drugs

^a^Grey literature reports that did not provide detailed description of research methods

^b^Did not specify duration/length of injecting heroin

### Sociodemographic characteristics of PWID

A total of *n* = 4622 PWID were assessed across 14 studies; in one study unit of analysis was country (Table 1). Over 95% of the sample were male. Most PWID were below the age of 35 and around half reported being married [49, 54, 58–61, 67–69]. Low levels of education were common, which was typically below 6 years of formal education [49, 54, 60, 61, 68, 69]. Five out of the 15 studies reported average monthly income which ranged from 3000–6900 Afghanis: equivalent to approximately US $60–138 around the study periods [49, 54, 60, 68, 69]. Having been refugee in the last 10 years was reported by 65%-92% of the participants in 6 studies [49, 54, 60, 61, 68, 69]. Only two studies reported homelessness among participants; in one study with a convenience sample of 463 male participants, only 4 reported homelessness [52], while another study with a convenience sample of 483 reported 23% of participant as homeless [61]. In the qualitative study with women who inject drugs, mean age was 42 years, all participants were ever married and had children [53]. Ninety percent had never been to school and the majority identified themselves as economically disadvantaged [53].

### Risk factors of IDU use (Q1)

Only one quantitative study using a cross-sectional design and convenience sample of 55 PWID examined the risk factors of IDU [66]. Joblessness (OR = 2.92; 95%CI: 1.20–7.11), starting drug use in another country (OR = 7.46;95%CI: 1.99–28.03), and previous history of incarceration (OR = 3.75;95%CI: 1.85–6.86) were significantly associated with IDU in multivariate binary logistic regression analysis [66]. According to two qualitative studies, participants reported physical effects (stronger kick) and cost as reasons for injecting drugs as they reported injecting to have the lowest daily cost compared to other routes of drug administration [53, 59]. Male PWID also reported fear of arrest as a reason for switching to injecting as they perceived it to be less publicly noticeable than smoking [59].

### Drug use characteristics and risk behaviours (Q2)

Eleven studies (10 quantitative and 2 qualitative) described the drug use characteristics of PWID [14, 49, 53, 54, 58, 59, 61, 64, 67–69]. Heroin was the most common drug used [14, 51, 54, 61, 67–69]. Some studies reported opium as the second most common drug used among PWID [14, 67, 68]. Polydrug use was common [49, 54, 61, 68, 69] with 20–77% of participants reporting mixing heroin with pheniramine maleate (Avil) [49, 54, 61, 69]. On average, age at which PWID initiated injecting drugs was 26 years based on four studies [14, 49, 54, 61]. In terms of place of drug consumption, male PWID reported public settings as common place of injecting and using drugs [51, 69] while for women, drug use usually took place at home with either spouse, friends, or neighbours [53].

Risk behaviours among PWID was reported by nine studies and common risk behaviours included needle/syringe sharing, re-using needles/syringes, paying for sex with a female, men who have sex with men, low condom use and re-injecting blood [14, 47, 49, 53, 54, 60, 61, 68, 69]. The proportion of PWID that reported ever needle/syringe sharing ranged from 17% in a convenience sample of 483 IDU recruited from harm reduction sites and areas where drug users congregate in Kabul [61] to 87% in a population-based survey [14]. In a qualitative study of IDU with 10 women from Kabul, sharing needles/syringes was reporting to be common, especially with a spouse [53]. Furthermore, three studies reported that 70–83% of PWID drew and re-injected their own blood [51, 54, 61]. The proportion of male participants who reported ever patronizing a female sex worker ranged from 40 to 76% and the proportion of male PWID ever having sex with a male was between 11 to 28% [49, 54, 60, 61, 68, 69]. Five studies reported condom use behaviour among PWID, of which four reported fewer than one-third of PWID ever using a condom [49, 54, 60, 61, 68, 69].

### Health and social burden of IDU (Q3)

Eight quantitative studies (6 cross-sectional, 1 observational cohort) examined the burden of infectious diseases among PWID with HIV and hepatitis C as the most common examined diseases [49, 54, 58, 60, 61, 67–69]. Point prevalence of HIV in studies that used confirmed biological measures ranged from 1.8% to 7.1% [49, 54, 58, 61, 68]. One observational cohort study reported an incidence rate of 1.5 cases per 100 person-year and median time to infection of 18.3 months [62]. Furthermore, a large (*n* = 1378) cross-sectional study in 2019 reported an HIV prevalence of 20.7% based on self-report [67]. Among those who tested positive for HIV, co-infection with hepatitis C virus (HCV) was very common [54, 58, 61]. Point prevalence of HCV based on biological measures ranged from 36.0% to 60.2% [49, 54, 58, 61, 68]. An observational cohort study reported an incidence rate of 35.1 cases per 100 person-year and median time to HCV infection of 9.3 months [62]. Hepatitis B prevalence ranged from 4.6% to 7.1% [49, 54, 61, 68], while syphilis prevalence ranged from 1.2% to 5.5% [60, 61, 68]. One study reported crude mortality rate among PWID (93.4/1000 person-year), 43.9% of which was due to overdose during an 18-month follow-up period [62]. Burden of mental health among PWID was only examined in one study with *n* = 83 participants recruited from an OST pilot programme in Kabul [58]; participants reported a mean number of 6.5 mental health symptoms. The two qualitative studies reported perceived psychological distress, infections, various physical health symptoms, and overdose among PWID [53, 59].

Only five studies examined the social burden of IDU among PWID, by reporting the burden of incarceration, criminalization, and perceived stigma. According to four quantitative studies, burden of incarceration among PWID ranged from 51.8% to 63.1% [49, 54, 58, 61]. The qualitative study conducted with 10 women who inject drugs reported experiences of community and intimate partner violence, economic hardships as well as marginalization [53]. Criminalization and fear of it were reported to be common among male PWID based on two studies [58, 59]. Furthermore, one qualitative study with male PWID reported perceived material deprivation and stigma at the family and community levels [59].

### Access to harm reduction and treatment services (Q4)

Nine studies (6 quantitative, 2 qualitative, 1 mixed methods) reported on access to harm reduction and treatment services [14, 46, 52, 53, 58, 61, 67–69]. National coverage of needle and syringe programmes (NSP) and opioid substitution therapy (OST) in Afghanistan was reported to be low based on an assessment of a programme funded by *The Global Fund* [65]. According to this study, the programme only covered 28% of the PWID population and average number of sterile needles/syringes provided per PWID per year was 157 compared to the 300 needles/syringes per PWID per year standard recommended by the World Health Organization [65]. Furthermore, harm reduction services provided by the *Afghanistan National Program for Control of AIDS, STI and Hepatitis*, only reached 14% of the PWID population, provided OST for 2%, and 50 sterile needles/syringes and 5 condoms were provided per PWID in 2019 [67]. Access to addiction treatment was reported to be low [52, 53] and difficulty accessing sterile needles/syringes was common [49, 53]. Pharmacies were reported as the usual place for obtaining sterile needles [51, 69] and in one study that examined service utilization among PWID, only 1 participant reported ever receiving syringes from a NSP [52]. Barriers to accessing harm reduction and treatment services included financial limitations, service capacity issues, fear of arrest, as well as stigma and discrimination [52, 53, 59].

Three studies reported HIV testing among PWID; the proportion of study participants reporting ever being tested ranged from high (82%) to low (22%) [14, 53, 67]. Knowledge about HIV was assessed by 4 studies, 3 of which reported low knowledge among PWID [50, 68, 69], while 1 study reported 60.2% of OST clients having correct HIV knowledge [58]. Only one study assessed OST programme retention rate with a follow-up period of 18 months [58]. The study reported a retention rate of 54.2% at 18 months, with reasons for lost to follow-up including imprisonment and migration among other reasons [58].

## Discussion

Results of this scoping review indicate that PWID in Afghanistan tend to be young (below the age of 35), married, and have low education. A high percentage of them reported being incarcerated, similar to trends among PWID in North America and elsewhere [18]. What is unique about PWID in Afghanistan is a large proportion reported previously being refugees in neighbouring countries of Iran and Pakistan, some of whom initiated drug use [14] and injecting behaviour in these countries [61, 64, 68]. In a study of Pakistani and Afghan refugee drug users, the authors found the latter group nearly three times less likely to have heard of HIV/AIDS and none of those ever sexually active reported ever using a condom compared to their Pakistani counterparts [70]. Thus, our findings have important implications for HIV and harm reduction programmes in countries hosting large numbers of vulnerable Afghan refugees. Our results demonstrated a sever lack of knowledge regarding risk factors of IDU in Afghanistan as only one study examined this with a small (*n* = 55) sample of male PWID. Previous incarceration was among one of the risk factors. This and a high prevalence of incarceration among PWID in Afghanistan suggests prisons may be an important setting for delivering harm reduction and addiction treatment services for frequently displaced and hard-to-reach PWID populations in Afghanistan.

Heroin first and opium second were the most common drugs consumed among PWID. Use of synthetic opioids and stimulants was relatively rare in comparison to global drug use trends [18]. Although this trend has remained constant according to a recent study of PWID in Afghanistan [67], there is need for ongoing research to monitor trends as synthetic drug markets have emerged in recent years in Afghanistan and in the neighbouring countries of Iran and Pakistan [4, 6]. Furthermore, polydrug use appears to be common among PWID in Afghanistan further warranting a need for ongoing monitoring of drug use patterns to inform evidence-based harm reduction and treatment programmes.

Engagement in risky sexual behaviours such as patronizing female sex workers, sex with a male, and low condom use was very common among male PWID in Afghanistan. Although comparison with other settings is difficult due to varying measurements and definitions, PWID in Afghanistan appear to have higher prevalence of sexual risk as 67–83% [60, 69] reported unprotected sex with a casual partner while prevalence of sexual risk was 36.7% in South Asia, 40.1% in the Middle East and North Africa, and 37.4% globally according to a 2017 systematic review [18]. Evidence of a network of high-risk groups (PWID, female sex workers, men who have sex with men) coupled with low condom use have important implications for widespread transmission of HIV, which is currently concentrated among drug users [30]. Therefore, it is important for future studies to assess risk behaviours among high-risk networks in Afghanistan. Ever sharing needles/syringes was very common among PWID in Afghanistan. Past 12-month prevalence of needle/syringe sharing was 87% among current injecting drug users according to a population-based survey [14], which is more than double the past 6–12-month global estimate of receptive needle/syringe sharing among PWID (32.8%) [37]. A nuanced risk behaviour reported among male PWID in Afghanistan was drawing and reinjecting one’s own blood [51, 54, 61]. Similar risk behaviours have been reported among PWID in Pakistan [71], Sub-Saharan Africa [72], and North America [73, 74], which is shown to be a risk factor for HIV transmission [73] and soft-tissue abscess [74]. This merits further examination to understand the patterns and impacts of this behaviour in terms of disease transmission.

There is limited information regarding the health burden of IDU among PWID in Afghanistan. The most common diseases studied were HIV and hepatitis with prevalence based on biological measures ranging from 1.8–7.1% for HIV, 36.0–60.2% for HCV, and 4.6–7.1% for hepatitis B virus (HBV). In comparison, the global prevalence of HIV, HCV, and HBV according to a 2017 systematic review was 17.8%, 52.3% and 9.1%, respectively [18]. The estimates for Afghanistan likely underestimate the true extent of HIV and hepatitis among PWID as they were based on small convenience samples that did not include women and rural PWID. According to a large study conducted with 1378 PWID across 8 cities, self-reported prevalence of HIV was 27% [67], further suggesting underestimation of the true burden of infectious diseases in this population. Information about mortality rates and comorbidity among PWID is limited as psychological disorders and other communicable diseases of concern (e.g., malaria and tuberculosis) [29] are not assessed to date. Moreover, except one observational cohort study conducted a decade ago [62], studies on fatal and non-fatal overdoses among PWID are non-existent. Information on the social burden of IDU is also limited in Afghanistan, particularly with regards to important indicators such as homelessness, financial problems, and systemic discrimination.

Access to harm reduction and addiction treatment is extremely limited among PWID in Afghanistan. Similar access barriers as reported by other PWID worldwide included financial limitations, capacity issues, as well as stigma and discrimination [75]. Of important note is fear of police harassment as it was both reported as a reason why PWID chose injecting as route of drug administration and as a barrier to accessing harm reduction programmes [53, 59]. This coupled with high incarceration rates among PWID indicate an opportunity to collaborate with law enforcement to expand provision of critical harm reduction programmes such as HIV testing and counselling, OST, NSP, and treatment referrals, which have been successfully implemented in the neighbour country, Iran [76–78] and have led to positive outcomes in different countries [79]. In addition to limited access to HIV testing and counselling, knowledge about HIV/AIDS is low among PWID in Afghanistan, which has important implications for HIV prevention and treatment programmes, particularly in the context of widespread needle/syringe sharing and sexual risk behaviours. While harm reduction programmes namely NSP, OST, and HIV testing and counselling have been implemented in Afghanistan and drug treatment programmes have been associated with positive outcomes (reduced drug use and criminal activity) [80, 81], our findings indicate lack of evaluation of treatment and harm reduction programmes for PWID.

### Moving forward with IDU research in Afghanistan

Illicit drug use patterns are highly gendered in Afghanistan, in terms of drug types, route of administration, and place of consumption [14, 47]. Women’s drug use is hidden and often happens at home whereas men commonly use drugs in public places. This has led to a gender gap in research as it has reportedly been difficult to recruit women PWID in research. This warrants the need to conduct research with women who inject drugs to better understand gender-based risk factors and barriers to treatment and harm reduction services. Furthermore, majority of the studies did not include rural populations, which according to a population-based survey in 2015, had drug use rates nearly 3 times higher than the urban populations [15]. As such, the extant literature does not highlight the true extent, nature, and impacts of IDU in Afghanistan. Lack of data on IDU has serious implications for implementation of evidence-based prevention and treatment interventions. Inadequate information on determinants and impacts of IDU and limited knowledge about service needs and barriers to care among PWID contributes to difficulties in garnering support for resource allocation towards uptake and expansion of evidence-based solutions and improvement of existing programmes serving these populations.

Research on IDU has dramatically decreased during the last decade in Afghanistan (Table 1), despite a growing trend in illicit drug availability and use [5, 13–15]. This warrants the need for continued research and surveillance of illicit drug use patterns and impact as well as evaluation of substance use care programmes. While conducting research in Afghanistan is difficult due to the complex environments as has been reported by studies included in this review [15, 53, 67, 69], it is critical for generating necessary evidence to identify and address the health care needs of vulnerable populations such as PWID. Potential solutions to overcome research barriers – to some extent – include greater resource mobilization towards health research and local capacity building through greater international collaborations [82, 83].

The present scoping review had some limitations. We restricted the search results to English language publications, which may have resulted in potential loss of relevant information. In addition, we missed reports that are not posted online as we did not seek additional information via personal communication with staff from relevant agencies. Nonetheless, there are several strengths to note. We developed a comprehensive and highly sensitive search strategy that was pretested. We searched several relevant grey literature databases following expert technical guidelines in a systematic fashion to allow replicability. Additionally, no restrictions were placed on publication date.

## Conclusions

This scoping review identified and mapped the extant literature on IDU in Afghanistan and revealed several important knowledge gaps. Overall, Afghan PWID have high levels of injecting and sexual risk behaviours compared to global estimates. High incarceration rates, displacement, and limited availability of harm reduction and treatment resources puts this population at great risk of carrying the burden of infectious diseases and other harms. A national, public health-oriented drug policy and substance use care programme should be a key long-term development goal for Afghanistan to promote uptake and expansion of evidence-based harm reduction and addiction treatment strategies. Development goals for Afghanistan should also include resource allocation for health research and local capacity building in order to address the need for ongoing and scientifically rigorous research, necessary for guiding priority setting for drug policy and substance use care. Future research should also address the current gender gap in IDU research.

The current humanitarian crisis due to Taliban occupation has worsened pre-existing risk environments including widespread poverty, displacement, and fragile healthcare systems. Several news reports have described rising illicit drug use and a shift towards punitive drug prevention and treatment strategies consisting of imprisonment and forced, unmedicated detoxification. There is an urgent need for international aid agencies and their partners to prioritize provision of harm reduction and evidence-based addiction treatment in Afghanistan.

## Supplementary Information

**Additional file 1. **Understanding injecting drug use in Afghanistan: A scoping review.

## Data Availability

Not applicable.

## Abbreviations

IDU: Injecting Drug Use
PWID: People Who Inject Drugs
JBI: Joanna Briggs Institute
PRISMA: Preferred Reporting Items for Systematic Reviews and Meta-Analyses
PRISMA-ScR: Preferred Reporting Items for Systematic Reviews and Meta-Analyses Extension for Scoping Reviews
HIV: Human Immunodeficiency Virus
HCV: Hepatitis C Virus
HBV: Hepatitis B Virus
OST: Opioid Substitution Therapy
NSP: Needle and Syringe Programme

## References

[1] Ward C, Byrd W (2004). Afghanistan’s opium drug economy.

[2] World Drug Report 2021 (United Nations publication, Sales No. E.21.XI.8). Available from: https://www.unodc.org/res/wdr2021/field/WDR21_Booklet_2.pdf

[3] United Nations Office on Drugs and Crime (UNDOC) (2021). Afghanistan opium survey 2021: Cultivation and production.

[4] European Monitoring Centre for Drugs and Drug Addiction (EMCDDA) (2020). Emerging evidence of Afghanistan’s role as a producer and supplier of ephedrine and methamphetamine.

[5] United Nations Office on Drugs and Crime (UNDOC) (2021). Drug situation in Afghanistan 2021: Latest findings and emerging threats.

[6] United Nations Office on Drugs and Crime (UNDOC) (2017). Afghanistan synthetic drugs situation assessment.

[7] National Statistics and Information Authority. Afghanistan Multidimensional Poverty Index 2016–2017. NSIA, Kabul; 2019. Available from: https://www.mppn.org/wp-content/uploads/2019/03/AFG_2019_vs9_online.pdf

[8] Crawford NC (2016). Cost of war: Update on the human costs of war for Afghanistan and Pakistan, 2001 to mid-2016.

[9] Panter-Brick C, Goodman A, Tol W, Eggerman M (2011). Mental health and childhood adversities: a longitudinal study in Kabul, Afghanistan. J Am Acad Child Adolesc Psychiatry.

[10] Scholte WF, Olff M, Ventevogel P, de Vries GJ, Jansveld E, Cardozo BL, Crawford CA (2004). Mental health symptoms following war and repression in eastern Afghanistan. JAMA.

[11] Rhodes T (2002). The ‘risk environment’: a framework for understanding and reducing drug-related harm. Int J Drug Policy.

[12] Collins AB, Boyd J, Cooper HL, McNeil R (2019). The intersectional risk environment of people who use drugs. Soc Sci Med.

[13] United Nations Office on Drugs and Crime (UNDOC) (2005). Afghanistan drug use survey 2005.

[14] United Nations Office on Drugs and Crime (UNDOC) (2009). Afghanistan drug use survey 2009.

[15] SGI-Global (2015). Afghanistan National Drug Use Survey 2015.

[16] United Nations Office on Drugs and Crime, World Drug Report 2017 (ISBN: 978–92–1–148291–1, eISBN: 978–92–1–060623–3, United Nations publication, Sales No. E.17.XI.6). Available from: https://www.unodc.org/wdr2017/field/Booklet_2_HEALTH.pdf

[17] Degenhardt L, Charlson F, Ferrari A, Santomauro D, Erskine H, Mantilla-Herrara A, Vos T (2018). The global burden of disease attributable to alcohol and drug use in 195 countries and territories, 1990–2016: a systematic analysis for the Global Burden of Disease Study 2016. Lancet Psychiatry.

[18] Degenhardt L, Peacock A, Colledge S, Leung J, Grebely J, Vickerman P, Larney S (2017). Global prevalence of injecting drug use and sociodemographic characteristics and prevalence of HIV, HBV, and HCV in people who inject drugs: a multistage systematic review. Lancet Glob Health.

[19] Novak SP, Kral AH (2011). Comparing injection and non-injection routes of administration for heroin, methamphetamine, and cocaine users in the United States. J Addict Dis.

[20] Strang J, Bearn J, Farrell M, Finch E, Gossop M, Griffiths P, Wolff K (1998). Route of drug use and its implications for drug effect, risk of dependence and health consequences. Drug Alcohol Rev.

[21] Darke S, Hall W (2003). Heroin overdose: research and evidence-based intervention. J Urban Health.

[22] Shrestha S, Stopka TJ, Hughto JM, Case P, Palacios WR, Reilly B, Green TC (2021). Prevalence and correlates of non-fatal overdose among people who use drugs: findings from rapid assessments in Massachusetts, 2017–2019. Harm Reduct J.

[23] Onyeka IN, Basnet S, Beynon CM, Tiihonen J, Föhr J, Kauhanen J (2016). Association between routes of drug administration and all-cause mortality among drug users. J Substance Use.

[24] Griffin N, Khoshnood K (2010). Opium trade, insurgency, and HIV/AIDS in Afghanistan: relationships and regional consequences. Asia Pacific J Public Health.

[25] World Health Organization (2019). The global health observatory: Afghanistan.

[26] The World Bank Group in Afghanistan (2021). Country update.

[27] Xinwei L (2019). The drug situation and the practice of drug control in Afghanistan. Himalayan Central Asian Stud.

[28] Todd CS, Nassiramanesh B, Stanekzai MR, Kamarulzaman A (2007). Emerging HIV epidemics in Muslim countries: assessment of different cultural responses to harm reduction and implications for HIV control. Curr HIV/AIDS Rep.

[29] Wallace MR, Hale BR, Utz GC, Olson PE, Earhart KC, Thornton SA, Hyams KC (2002). Endemic infectious diseases of Afghanistan. Clin Infect Dis.

[30] Mumtaz GR, Riedner G, Abu-Raddad LJ (2014). The emerging face of the HIV epidemic in the Middle East and North Africa. Curr Opin HIV AIDS.

[31] Gutlove P, Thompson G, Russell JH (2004). Health, Human Security, and Social Reconstruction in Afghanistan. Beyond Reconstruction in Afghanistan.

[32] Grebely J, Larney S, Peacock A, Colledge S, Leung J, Hickman M, Degenhardt L (2019). Global, regional, and country-level estimates of hepatitis C infection among people who have recently injected drugs. Addiction.

[33] Rashti R, Sharafi H, Alavian SM, Moradi Y, Mohamadi Bolbanabad A, Moradi G (2020). Systematic Review and Meta-Analysis of Global Prevalence of HBsAg and HIV and HCV Antibodies among People Who Inject Drugs and Female Sex Workers. Pathogens (Basel, Switzerland).

[34] Larney S, Leung J, Grebely J, Hickman M, Vickerman P, Peacock A, Degenhardt L (2020). Global systematic review and ecological analysis of HIV in people who inject drugs: National population sizes and factors associated with HIV prevalence. Int J Drug Policy.

[35] Arum C, Fraser H, Artenie AA, Bivegete S, Trickey A, Alary M, Strathdee SA (2021). Homelessness, unstable housing, and risk of HIV and hepatitis C virus acquisition among people who inject drugs: a systematic review and meta-analysis. Lancet Public Health.

[36] Mathers BM, Degenhardt L, Ali H, Wiessing L, Hickman M, Mattick RP, Strathdee SA (2010). HIV prevention, treatment, and care services for people who inject drugs: a systematic review of global, regional, and national coverage. Lancet.

[37] Tran LT, Peacock A, Colledge S, Memedovic S, Grebely J, Leung J, Larney S, Trickey A, Stone J, Vickerman P, Hickman M, Degenhardt L (2020). Injecting risk behaviours amongst people who inject drugs: A global multi-stage systematic review and meta-analysis. Int J Drug Policy.

[38] Munn Z, Peters MD, Stern C, Tufanaru C, McArthur A, Aromataris E (2018). Systematic review or scoping review? Guidance for authors when choosing between a systematic or scoping review approach. BMC Med Res Methodol.

[39] Arksey H, O'Malley L (2005). Scoping studies: towards a methodological framework. Int J Soc Res Methodol.

[40] Peters MD, Marnie C, Tricco AC, Pollock D, Munn Z, Alexander L, Khalil H (2020). Updated methodological guidance for the conduct of scoping reviews. JBI Evidence Synthesis.

[41] Levac D, Colquhoun H, O'Brien KK (2010). Scoping studies: advancing the methodology. Implement Sci.

[42] Tricco AC, Lillie E, Zarin W, O'Brien KK, Colquhoun H, Levac D, Straus SE (2018). PRISMA extension for scoping reviews (PRISMA-ScR): checklist and explanation. Ann Intern Med.

[43] Paez A (2017). Gray literature: An important resource in systematic reviews. J Evid Based Med.

[44] Mahood Q, Van Eerd D, Irvin E (2014). Searching for grey literature for systematic reviews: challenges and benefits. Res Synthesis Methods.

[45] Degenhardt L, Gibson G, Leung J, Kumvaj M, Lareney S (2016). Searching the grey literature to access research on illicit drug use, HIV, and viral hepatitis.

[46] Burrows D, McCallum L, Parsons D Falkenberry H. Global Summary of Findings of an Assessment of HIV Service Packages for Key Populations in Six Regions. APMG Health, Washington, DC; 2019. Available from: https://www.theglobalfund.org/media/9753/core_hivservicesforkeypopulationssixregions_review_en.pdf

[47] UNODC, Impacts of Drug Use on Users and Their Families in Afghanistan, 2014. Available from: https://www.unodc.org/documents/data-and-analysis/Studies/Impacts_Study_2014_web.pdf

[48] Bautista CT, Todd CS, Abed AM, Botros BA, Strathdee SA, Earhart KC, Scott PT (2010). Effects of duration of injection drug use and age at first injection on HCV among IDU in Kabul. Afghanistan J Public Health.

[49] Todd CS, Abed AM, Strathdee SA, Scott PT, Botros BA, Safi N, Earhart KC (2007). HIV, hepatitis C, and hepatitis B infections and associated risk behavior in injection drug users, Kabul. Afghanistan Emerg Infect Dis.

[50] Todd CS, Abed A, Strathdee SA, Scott PT, Botros BA, Safi N, Earhart KC (2007). Association between expatriation and HIV awareness and knowledge among injecting drug users in Kabul, Afghanistan: A cross-sectional comparison of former refugees to those remaining during conflict. Confl Heal.

[51] Todd CS, Abed AM, Scott PT, Botros BA, Safi N, Earhart KC, Strathdee SA (2008). Correlates of receptive and distributive needle sharing among injection drug users in Kabul, Afghanistan. Am J Drug Alcohol Abuse.

[52] Todd CS, Abed AM, Scott PT, Safi N, Earhart KC, Strathdee SA (2009). A cross-sectional assessment of utilization of addiction treatment among injection drug users in Kabul. Afghanistan Subst Use Misuse.

[53] Middle East and North Africa Harm Reduction Association (MENAHRA) (2013). Women injecting drug users in the Middle East and North Africa Region (MENA).

[54] Nasir A, Todd CS, Stanekzai MR, Bautista CT, Botros BA, Scott PT, Tjaden J (2011). Prevalence of HIV, hepatitis B and hepatitis C and associated risk behaviours amongst injecting drug users in three Afghan cities. Int J Drug Policy.

[55] Nasir A, Todd CS, Stanekzai MR, Bautista CT, Botros BA, Scott PT, Tjaden J (2011). Implications of hepatitis C viremia vs, antibody alone on transmission among male injecting drug users in three Afghan cities. Int J Infect Dis.

[56] Sanders-Buell E, Bose M, Nasir A, Todd CS, Stanekzai MR, Tovanabutra S, McCutchan FE (2010). Distinct circulating recombinant HIV-1 strains among injecting drug users and sex workers in Afghanistan. AIDS Res Hum Retroviruses.

[57] Sanders-Buell E, Rutvisuttinunt W, Todd CS, Nasir A, Bradfield A, Lei E, Tovanabutra S (2013). Hepatitis C genotype distribution and homology among geographically disparate injecting drug users in Afghanistan. J Med Virol.

[58] Ruiseñor-Escudero H, Vu A, Wirtz AL, Familiar-Lopez I, Berry M, Mfochive I, Burnham G (2015). Cross-sectional assessments of participants’ characteristics and loss to follow-up in the first Opioid Substitution Therapy Pilot Program in Kabul. Afghanistan Harm Reduc J.

[59] Todd CS, Stibich MA, Stanekzai MR, Rasuli MZ, Bayan S, Wardak SR, Strathdee SA (2009). A qualitative assessment of injection drug use and harm reduction programmes in Kabul, Afghanistan: 2006–2007. Int J Drug Policy.

[60] Todd CS, Nasir A, Stanekzai MR, Abed AM, Strathdee SA, Bautista CT, Tjaden J (2010). Prevalence and correlates of syphilis and condom use among male injection drug users in four Afghan cities. Sex Transm Dis.

[61] Todd CS, Nasir A, Stanekzai MR, Fiekert K, Rasuli MZ, Vlahov D, Strathdee SA (2011). Prevalence and correlates of HIV, syphilis, and hepatitis B and C infection and harm reduction program use among male injecting drug users in Kabul, Afghanistan: A cross-sectional assessment. Harm Reduct J.

[62] Todd CS, Nasir A, Stanekzai MR, Fiekert K, Sipsma HL, Vlahov D, Strathdee SA (2015). Hepatitis C and HIV incidence and harm reduction program use in a conflict setting: an observational cohort of injecting drug users in Kabul. Afghanistan Harm Reduc J.

[63] Todd CS, Nasir A, Stanekzai MR, Fiekert K, Sipsma HL, Strathdee SA, Vlahov D (2016). Impact of conflict and displacement on risk behaviours amongst people who inject drugs in Kabul, Afghanistan. Int J Drug Policy.

[64] Vogel M, Tschakarjan S, Maguet O, Vandecasteele O, Kinkel T, Dˇrsteler-MacFarland K (2012). Mental health among opiate users in Kabul: a pilot study from the Medecins du Monde Harm Reduction Programme. Intervention.

[65] Burrows D, Falkenberry H, McCallum L, Parsons D, Ngoksin E, Zhao J, Kunii O (2021). Design, implementation, and monitoring of HIV service packages for people who inject drugs: An assessment of programs supported by the Global Fund in 46 countries. Int J Drug Policy.

[66] Rasekh H, Naimi HM, Mousavi SH. Prevalence and risk factors of hepatitis B, hepatitis C and HIV viruses among people who use drugs (PWUD) in Kabul, health care facilities. Hepat Mon. 2019;19(7):e84298.

[67] Rasheed A, Sharifi H, Wesson P, Pashtoon SJ, Tavakoli F, Ghalekhani N, Mirzazadeh A (2022). Mapping and population size estimates of people who inject drugs in Afghanistan in 2019: Synthesis of multiple methods. PLoS ONE.

[68] Ruiseñor-Escudero H, Wirtz AL, Berry M, Mfochive-Njindan I, Paikan F, Yousufi HA, Yadav RS, Burnham G, Vu A (2014). Risky behavior and correlates of HIV and Hepatitis C virus infection among people who inject drugs in three cities in Afghanistan. Drug Alcohol Depend.

[69] World Bank. 2008. Mapping and Situation Assessment of Key Populations at High Risk of HIV in Three Cities of Afghanistan. South Asia human development sector series;no. 23. Washington, DC. © World Bank. https://openknowledge.worldbank.org/handle/10986/17938 License: CC BY 3.0 IGO. Available from: https://openknowledge.worldbank.org/bitstream/handle/10986/17938/437330NWP0Box31gAfghanistanApril708.pdf?sequence=1&isAllowed=y

[70] Zafar T, Brahmbhatt H, Imam G, Hassan SU, Strathdee SA (2003). HIV knowledge and risk behaviors among Pakistani and Afghani drug users in Quetta, Pakistan. JAIDS J Acquir Immune Defic Syndr.

[71] Kuo I, Galai N, Thomas DL, Zafar T, Ahmed MA, Strathdee SA (2006). High HCV seroprevalence and HIV drug use risk behaviors among injection drug users in Pakistan. Harm Reduct J.

[72] Asher AK, Hahn JA, Couture MC, Maher K, Page K (2013). People who inject drugs, HIV risk, and HIV testing uptake in sub-Saharan Africa. J Assoc Nurses AIDS Care JANAC.

[73] Bruneau J, Daniel M, Abrahamowicz M, Zang G, Lamothe F, Vincelette J (2011). Trends in human immunodeficiency virus incidence and risk behavior among injection drug users in Montreal, Canada: a 16-year longitudinal study. Am J Epidemiol.

[74] Murphy EL, DeVita D, Liu H, Vittinghoff E, Leung P, Ciccarone DH, Edlin BR (2001). Risk factors for skin and soft-tissue abscesses among injection drug users: a case-control study. Clin Infect Dis.

[75] Harm Reduction International (2012). The global state of harm reduction: Towards an integrated response.

[76] Ohiri K, Claeson M, Rassaghi E, Nassirimanesh B, Afshar P, Power R (2006). HIV/AIDS prevention among injection drug users: Learning from harm reduction in Iran.

[77] Saberi Zafarghandi MB, Jadidi M, Khalili N (2015). Iran's Activities on Prevention, Treatment and Harm Reduction of Drug Abuse. Int J High Risk Behav Addict.

[78] Shahbazi M, Farnia M, Rahmani K, Moradi G (2014). Trend of HIV/AIDS Prevalence and Related Interventions Administered in Prisons of Iran -13 Years' Experience. Iran J Public Health.

[79] Kamarulzaman A, Reid SE, Schwitters A, Wiessing L, El-Bassel N, Dolan K, Moazen B, Wirtz AL, Verster A, Altice FL (2016). Prevention of transmission of HIV, hepatitis B virus, hepatitis C virus, and tuberculosis in prisoners. Lancet (London Engl).

[80] Courser M, Johnson K, Abadi MH, Shamblen SR, Young L, Thompson K, Browne T (2013). Building an evidence base for drug abuse treatment in Afghanistan: Lessons learned and implications for future research. Int J Prev Treatment Subs Use Dis.

[81] Shamblen SR, Courser M, Young L, Schweinhart A, Shepherd C, Morales B, Redpath B. The Efficacy of Drug Treatment in Afghanistan: Overall Results and Implications From a New Evaluation. Int J Mental Health Addiction. 2022;20:541–52. 10.1007/s11469-020-00382-1.

[82] El Achi N, Papamichail A, Rizk A, Lindsay H, Menassa M, Abdul-Khalek RA, Ekzayez A, Dewachi O, Patel P (2019). A conceptual framework for capacity strengthening of health research in conflict: the case of the Middle East and North Africa region. Glob Health.

[83] Mansour R, Naal H, Kishawi T, Achi NE, Hneiny L, Saleh S (2021). Health research capacity building of health workers in fragile and conflict-affected settings: a scoping review of challenges, strengths, and recommendations. Health Res Policy Syst.

