# Supplementary material for: Understanding injecting drug use in Afghanistan: A scoping review
Source: Subst Abuse Treat Prev Policy. 2022 Sep 19;17:65. doi: 10.1186/s13011-022-00491-1 (PMC9484158; doi:10.1186/s13011-022-00491-1)
Supplement: Supplementary file 1 — Additional file 1. Understanding injecting drug use in Afghanistan: A scoping review. [file 13011_2022_491_MOESM1_ESM.docx]

**Additional File 1:** Understanding Injecting Drug Use in Afghanistan: A Scoping Review

This document provides additional information on the methodology to support the results for the scoping review reported in the main text of the manuscript. This document also contains the Preferred Reporting Items for Systematic Reviews and Meta-Analyses extension for Scoping Reviews (PRISMA-ScR) checklist.

Supplementary Appendix A (Additional Details on the Methodology) Pages 2-7

Supplementary Appendix B (PRISMA-ScR Checklist) Pages 8-10

**Supplementary Appendix A: Additional Details on the Methodology**

**Bibliographic Database Search Strategies for Peer-Reviewed Literature**

The electronic search strategies operationalized in each of the databases are presented in Table S1.

**Table S1: Search strategies operationalized in Embase, Global Health, Medline, PsycINFO, and Web of Science**

| Embase | 1. exp intravenous drug abuse/ or exp intravenous drug administration/  2. exp injection drug user/  3. (inject* drug* or inject* substance*).mp.  4. (intravenous drug* or intravenous substance*).mp.  5. exp needle sharing/  6. *Pharmaceutical Preparations/  7. (IDU or IDUs or IVDU or IVDUs or PWID or PWIDs).mp.  8. ("needle* exchang*" or "syringe* exchang*").mp.  9. ("needle* shar*" or "syringe* shar*" or "needle/syringe sharing").mp.  10. (("shar*" or "re-using" or "reusing") adj2 "inject* equipment*").mp.  11. "people who inject".mp.  12. "person$ who inject".mp.  13. ((substance* or drug* or narcot* or stimulant* or cocaine or crack or methamphetamin* or benzodiazepin* or barbiturat* or ketamin* or hallucinogen* or opiat* or opioid* or opium or heroin* or fentanyl or carfentanil or carfentanyl or Wildnil or morphin* or morfin* or diamorphin* or "crystal meth" or "n methylamphetamine" or methadone or buprenorphine or hydromorphone) adj3 (inject* or "shoot* up" or "shot up" or intravenous* or parenteral*)).ti,ab,kw,kf,fs.  14. Afghanistan.mp. or exp Afghanistan/  15. exp Afghan/ or Afghan*.mp.  16. South Asia.ti,ab,kf.  17. South East Asia.ti,ab,kf.  18. Southeastern Asia.ti,ab,kf.  19. Central Asia.ti,ab,kf.  20. Middle East.ti,ab,kf.  21. 1 or 2 or 3 or 4 or 5 or 6 or 7 or 8 or 9 or 10 or 11 or 12 or 13  22. 14 or 15 or 16 or 17 or 18 or 19 or 20  23. 21 and 22  24. limit 23 to (human and english language) |
| --- | --- |
| Global Health | 1. (inject* drug* or inject* substance*).mp.  2. (intravenous drug* or intravenous substance*).mp.  3. (IDU or IDUs or IVDU or IVDUs or PWID or PWIDs).mp.  4. needle sharing/  5. (injecting drug abuse or injecting drug users).sh.  6. ("needle* exchang*" or "syringe* exchang*").mp.  7. ("needle* shar*" or "syringe* shar*" or "needle/syringe sharing").mp.  8. (("shar*" or "re-using" or "reusing") adj2 "inject* equipment*").mp.  9. "people who inject".mp.  10. "person$ who inject".mp.  11. ((substance* or drug* or narcot* or stimulant* or cocaine or crack or methamphetamin* or benzodiazepin* or barbiturat* or ketamin* or hallucinogen* or opiat* or opioid* or opium or heroin* or fentanyl carfentanil or carfentanyl or Wildnil or morphin* or morfin* or diamorphin* or "crystal meth" or "n methylamphetamine" or methadone or buprenorphine or hydromorphone) adj3 (inject* or "shoot* up" or "shot up" or intravenous* or parenteral*)).ab,ti,hw.  12. Afghanistan.mp. or Afghanistan/  13. Afghan*.mp.  14. South Asia.ti,ab,hw.  15. South East Asia.ti,ab,hw.  16. Southeastern Asia.ti,ab,hw.  17. Central Asia.ti,ab,hw.  18. Middle East.ti,ab,hw.  19. 1 or 2 or 3 or 4 or 5 or 6 or 7 or 8 or 9 or 10 or 11  20. 12 or 13 or 14 or 15 or 16 or 17 or 18  21. 19 and 20  22. limit 21 to english language |
| Medline^*^ | 1. inject* drug*.mp.  2. exp Substance Abuse, Intravenous/ or inject* substance*.mp.  3. intravenous drug*.mp.  4. intravenous substance*.mp.  5. IDU.mp.  6. IDUs.mp.  7. PWID.mp.  8. IVDU.mp.  9. ("needle* exchang*" or "needle/syringe sharing").mp.  10. exp Needle Sharing/ or "syringe* exchang*".mp. or *Pharmaceutical Preparations/  11. "syringe* shar*".mp.  12. Afghanistan.mp. or exp Afghanistan/  13. Afghan*.mp.  14. South Asia.ti,ab,kf.  15. Southeastern Asia.ti,ab,kf.  16. South East Asia.ti,ab,kf.  17. Central Asia.ti,ab,kf.  18. Middle East.ti,ab,kf.  19. "people who inject".mp.  20. "person$ who inject".mp.  21. "needle* shar*".mp.  22. IVDUs.mp.  23. PWIDs.mp.  24. ((substance* or drug* or narcot* or stimulant* or cocaine or crack or methamphetamin* or benzodiazepin* or barbiturat* or ketamin* or hallucinogen* or opiat* or opioid* or opium or heroin* or fentanyl or carfentanil or carfentanyl or Wildnil or morphin* or morfin* or diamorphin* or "crystal meth" or "n methylamphetamine" or methadone or buprenorphine or hydromorphone) adj3 (inject* or "shoot* up" or "shot up" or intravenous* or parenteral*)).ti,ab,kw,kf,fs.  25. (("shar*" or "re-using" or "reusing") adj2 "inject* equipment*").mp.  26. 1 or 2 or 3 or 4 or 5 or 6 or 7 or 8 or 9 or 10 or 11 or 19 or 20 or 21 or 22 or 23 or 24 or 25  27. 12 or 13 or 14 or 15 or 16 or 17 or 18  28. 26 and 27  29. limit 28 to (english language and humans) |
| PyscINFO | 1. ((substance* or drug* or narcot* or stimulant* or cocaine or crack or methamphetamin* or benzodiazepin* or barbiturat* or ketamin* or hallucinogen* or opiat* or opioid* or opium or heroin* or fentanyl or carfentanil or carfentanyl or Wildnil or morphin* or morfin* or diamorphin* or "crystal meth" or "n methylamphetamine" or methadone or buprenorphine or hydromorphone) adj3 (inject* or "shoot* up" or "shot up" or intravenous* or parenteral*)).ti,ab,hw.  2. (inject* drug* or inject* substance*).mp.  3. exp Drug Self Administration/  4. (intravenous drug* or intravenous substance*).mp.  5. (IDU or IDUs or IVDU or IVDUs or PWID or PWIDs).mp.  6. exp Needle Sharing/  7. exp Intravenous Drug Usage/  8. ("needle* exchang*" or "syringe* exchang*").mp.  9. ("needle* shar*" or "syringe* shar*" or "needle/syringe sharing").mp.  10. (("shar*" or "re-using" or "reusing") adj2 "inject* equipment*").mp.  11. "people who inject".mp.  12. "person$ who inject".mp.  13. Afghanistan.mp.  14. Afghan*.mp.  15. South Asia.ti,ab,tw.  16. South East Asia.ti,ab,tw.  17. Southeastern Asia.ti,ab,tw.  18. Central Asia.ti,ab,tw.  19. Middle East.ti,ab,tw.  20. 1 or 2 or 3 or 4 or 5 or 6 or 7 or 8 or 9 or 10 or 11 or 12  21. 13 or 14 or 15 or 16 or 17 or 18 or 19  22. 20 and 21  23. limit 22 to (human and english language) |
| Web of Science | 1. **(((((((((((((((((ALL=(inject* drug*)) OR ALL=(inject* substance*)) OR ALL=(intravenous drug*)) OR ALL=(intravenous substance*)) OR ALL=("people who inject")) OR ALL=("person$ who inject")) OR ALL=("IDU")) OR ALL=("IDUs")) OR ALL=("IVDU")) OR ALL=("IVDUs")) OR ALL=("PWID")) OR ALL=("PWIDs")) OR ALL=("needl* shar*")) OR ALL=("needl* exchang*")) OR ALL=("syringe* shar*")) OR ALL=("syringe* exchang*")) OR ALL=("pharmaceutical preparations")) OR ALL=("needle/syringe sharing")** 2. **(substance* or drug* or narcot* or stimulant* or cocaine or crack or methamphetamin* or benzodiazepin* or barbiturat* or ketamin* or hallucinogen* or opiat* or opioid* or opium or heroin* or fentanyl or morphin* or morfin* or diamorphin* or "crystal meth" or "n methylamphetamine" or methadone or buprenorphine or hydromorphone or Carfentanil or carfentanil or wildnis) NEAR/3 (inject* or "shoot* up" or “shot up” or parenteral* or intravenous)** (Keyword Plus ®) or **(substance* or drug* or narcot* or stimulant* or cocaine or crack or methamphetamin* or benzodiazepin* or barbiturat* or ketamin* or hallucinogen* or opiat* or opioid* or opium or heroin* or fentanyl or morphin* or morfin* or diamorphin* or "crystal meth" or "n methylamphetamine" or methadone or buprenorphine or hydromorphone or Carfentanil or carfentanil or wildnis) NEAR/3 (inject* or "shoot* up" or “shot up” or parenteral* or intravenous)** (Title) or **(substance* or drug* or narcot* or stimulant* or cocaine or crack or methamphetamin* or benzodiazepin* or barbiturat* or ketamin* or hallucinogen* or opiat* or opioid* or opium or heroin* or fentanyl or morphin* or morfin* or diamorphin* or "crystal meth" or "n methylamphetamine" or methadone or buprenorphine or hydromorphone or Carfentanil or carfentanil or wildnis) NEAR/3 (inject* or "shoot* up" or “shot up” or parenteral* or intravenous)** (Author Keywords)or **(substance* or drug* or narcot* or stimulant* or cocaine or crack or methamphetamin* or benzodiazepin* or barbiturat* or ketamin* or hallucinogen* or opiat* or opioid* or opium or heroin* or fentanyl or morphin* or morfin* or diamorphin* or "crystal meth" or "n methylamphetamine" or methadone or buprenorphine or hydromorphone or Carfentanil or carfentanil or wildnis) NEAR/3 (inject* or "shoot* up" or “shot up” or parenteral* or intravenous)** (Abstract) 3. **ALL=(("shar*" or "re-using" or "reusing") AND ("inject* equipment*"))** 4. **(((((TI=(("South Asia" or "Southeastern Asia" or "South East Asia" or "Central Asia" or "Middle East"))) OR AB=(("South Asia" or "Southeastern Asia" or "South East Asia" or "Central Asia" or "Middle East"))) OR AK=(("South Asia" or "Southeastern Asia" or "South East Asia" or "Central Asia" or "Middle East"))) OR KP=(("South Asia" or "Southeastern Asia" or "South East Asia" or "Central Asia" or "Middle East"))) OR ALL=(Afghanistan)) OR ALL=(Afghan*)** 5. **#1 OR #2 OR #3** 6. **#4 AND #5 and English (**Languages) |

^*^Search strategy was initially developed using Medline and then replicated in other databases.

**Search Strategies for Grey Literature**

Twelve online databases and websites consisting of international, government, harm reduction, HIV, and substance use organizations were searched for additional information. These databases have been identified as important sources of information on injecting drug use.^1^ The electronic search strategies operationalized in each database are presented in Table S2.

**Table S2: Search strategies operationalized in grey literature databases**

| Database Name and Link | Search Details | Number of Records Found | Number of Records Kept |
| --- | --- | --- | --- |
| Drug Policy Alliance   - <http://www.drugpolicy.org/library> | Search date: March 19, 2022  Searched website using advanced google search option.  Search terms: "inject drug" AND “Afghanistan” | 9 | 0 |
| Harm Reduction International   - <https://www.hri.global/> - Resource library | Search date: March 20, 2022  Searched website using advanced google search option.  Search terms: "inject drug" AND “Afghanistan”  Explored resource library using term “Afghanistan” | 9  19 | 0  0 |
| International AIDS Society Conference   - <https://www.abstract-archive.org/> | Search date: March 20, 2022  Explored abstract archives using term “Afghanistan” | 69 | 0 |
| International Drug Policy Consortium   - <https://idpc.net/> - Publications Library | Search date: March 19, 2022  Searched website using advanced google search option.  Search terms: "inject drug" AND “Afghanistan”  Searched publications library using term “Afghanistan” | 7  60 | 0  0 |
| International Narcotics Control Board (INCB)   - <http://www.incb.org/incb/index.html> | Search date: March 19, 2022  Searched website using advanced google search option.  Search terms: “inject drug” AND “Afghanistan” | 90 | 0 |
| The Middle East and North Africa Harm Reduction Association (MENAHRA)   - <https://www.menahra.org/en/> | Search date: March 20, 2022  Searched website using term “Afghanistan” | 38 | 1 |
| Ministry of Public Health Afghanistan   - Publication section - Reports | Search date: March 19, 2022  Explored publications section  Explored surveillance reports | 4  52 | 0  0 |
| International Association of Substance Abuse Librarians & Information Specialists   - <https://salis.org/> | Search date: March 20, 2022  Searched website using advanced google search option.  Search terms: "inject drug" AND “Afghanistan” | 2 | 0 |
| World Bank   - Research and Publications | Search date: March 20, 2022  Explored research and publications section using term “inject drug” | 90 | 1 |
| United Nations Office on Drugs and Crime (UNDOC)   - Publications - World Drug Reports | Search date: March 19, 2022  Explored all publications to date in the publications section  Explored all published (1997-2021) reports | 31  24 | 2  0 |
| UNAIDS   - <https://www.unaids.org/en> | Search date: March 21, 2022  Searched website using term “Afghanistan” | 105 | 0 |
| World Health Organization (WHO) libraries   - IRIS   <https://apps.who.int/iris/>   - WHOLIS   <https://kohahq.searo.who.int/>   - Global Index Medicus   <https://www.globalindexmedicus.net/> | Search date: March 21, 2022  Searched IRIS library using advanced filters and search terms: “inject drug” and “Afghanistan”  Searched WHOLIS library using advanced search option and search terms: “inject drug” AND “Afghanistan  Searched global Index Medicus library using advanced search option and search terms: “inject drug” AND “Afghanistan | 2  0  0 | 0  0  0 |

**^1^**Degenhardt, L., Gibson, G., Leung, J., Kumvaj, M, & Lareney, S. (2016). Searching the grey literature to access research on illicit drug use, HIV, and viral hepatitis. Available from: <https://ndarc.med.unsw.edu.au/sites/default/files/ndarc/resources/Technical%20report%20number%20334.pdf>

**Supplementary Appendix B**

**Preferred Reporting Items for Systematic Reviews and Meta-Analyses extension for Scoping Reviews (PRISMA-ScR) Checklist**

| **SECTION** | **ITEM** | **PRISMA-ScR CHECKLIST ITEM** | **REPORTED ON PAGE #** |
| --- | --- | --- | --- |
| **TITLE** | | | |
| Title | 1 | Identify the report as a scoping review. | Title Page, P. 1 |
| **ABSTRACT** | | | |
| Structured summary | 2 | Provide a structured summary that includes (as applicable): background, objectives, eligibility criteria, sources of evidence, charting methods, results, and conclusions that relate to the review questions and objectives. | Abstract, P. 1 |
| **INTRODUCTION** | | | |
| Rationale | 3 | Describe the rationale for the review in the context of what is already known. Explain why the review questions/objectives lend themselves to a scoping review approach. | Introduction (paragraphs 4 & 5), P. 2 |
| Objectives | 4 | Provide an explicit statement of the questions and objectives being addressed with reference to their key elements (e.g., population or participants, concepts, and context) or other relevant key elements used to conceptualize the review questions and/or objectives. | Introduction (paragraph 5), P. 2 |
| **METHODS** | | | |
| Protocol and registration | 5 | Indicate whether a review protocol exists; state if and where it can be accessed (e.g., a Web address); and if available, provide registration information, including the registration number. | Methods (paragraph 1), P. 2-3 |
| Eligibility criteria | 6 | Specify characteristics of the sources of evidence used as eligibility criteria (e.g., years considered, language, and publication status), and provide a rationale. | Methods (Inclusion and exclusion criteria), P. 3-4 |
| Information sources* | 7 | Describe all information sources in the search (e.g., databases with dates of coverage and contact with authors to identify additional sources), as well as the date the most recent search was executed. | Methods (Literature review and data sources), P. 3 |
| Search | 8 | Present the full electronic search strategy for at least 1 database, including any limits used, such that it could be repeated. | Supplementary Appendix A (Table S1 & Table S2) |
| Selection of sources of evidence† | 9 | State the process for selecting sources of evidence (i.e., screening and eligibility) included in the scoping review. | Methods (Data extraction, paragraph 1), P. 4 |
| Data charting process‡ | 10 | Describe the methods of charting data from the included sources of evidence (e.g., calibrated forms or forms that have been tested by the team before their use, and whether data charting was done independently or in duplicate) and any processes for obtaining and confirming data from investigators. | Methods (Data extraction, paragraph 2), P. 4 |
| Data items | 11 | List and define all variables for which data were sought and any assumptions and simplifications made. | Methods (Collecting, summarizing, and reporting results), P. 4 |
| Critical appraisal of individual sources of evidence§ | 12 | If done, provide a rationale for conducting a critical appraisal of included sources of evidence; describe the methods used and how this information was used in any data synthesis (if appropriate). | Not applicable |
| Synthesis of results | 13 | Describe the methods of handling and summarizing the data that were charted. | Methods (Collecting, summarizing, and reporting results), P. 4 |
| **RESULTS** | | | |
| Selection of sources of evidence | 14 | Give numbers of sources of evidence screened, assessed for eligibility, and included in the review, with reasons for exclusions at each stage, ideally using a flow diagram. | Results (Characteristics of included studies), P. 4 & Figure 1 |
| Characteristics of sources of evidence | 15 | For each source of evidence, present characteristics for which data were charted and provide the citations. | Results (Characteristics of included studies), P. 4 & Table 1 |
| Critical appraisal within sources of evidence | 16 | If done, present data on critical appraisal of included sources of evidence (see item 12). | Not Applicable |
| Results of individual sources of evidence | 17 | For each included source of evidence, present the relevant data that were charted that relate to the review questions and objectives. | Results (Risk factors of IDU; Drug use characteristics and risk behaviours; Health and social burden of IDU; Access to harm reduction and treatment services), P. 8-9 |
| Synthesis of results | 18 | Summarize and/or present the charting results as they relate to the review questions and objectives. | Results (Risk factors of IDU; Drug use characteristics and risk behaviours; Health and social burden of IDU; Access to harm reduction and treatment services), P. 8-9 |
| **DISCUSSION** | | | |
| Summary of evidence | 19 | Summarize the main results (including an overview of concepts, themes, and types of evidence available), link to the review questions and objectives, and consider the relevance to key groups. | Discussion (paragraphs 1-7), P. 9-11 |
| Limitations | 20 | Discuss the limitations of the scoping review process. | Discussion (paragraph 8), P. 11 |
| Conclusions | 21 | Provide a general interpretation of the results with respect to the review questions and objectives, as well as potential implications and/or next steps. | Conclusions, P. 11 |
| **FUNDING** | | | |
| Funding | 22 | Describe sources of funding for the included sources of evidence, as well as sources of funding for the scoping review. Describe the role of the funders of the scoping review. | Declarations (Funding), P. 11 |

JBI = Joanna Briggs Institute; PRISMA-ScR = Preferred Reporting Items for Systematic reviews and Meta-Analyses extension for Scoping Reviews.

* Where *sources of evidence* (see second footnote) are compiled from, such as bibliographic databases, social media platforms, and Web sites.

† A more inclusive/heterogeneous term used to account for the different types of evidence or data sources (e.g., quantitative and/or qualitative research, expert opinion, and policy documents) that may be eligible in a scoping review as opposed to only studies. This is not to be confused with *information sources* (see first footnote).

‡ The frameworks by Arksey and O’Malley (6) and Levac and colleagues (7) and the JBI guidance (4, 5) refer to the process of data extraction in a scoping review as data charting*.*

§ The process of systematically examining research evidence to assess its validity, results, and relevance before using it to inform a decision. This term is used for items 12 and 19 instead of "risk of bias" (which is more applicable to systematic reviews of interventions) to include and acknowledge the various sources of evidence that may be used in a scoping review (e.g., quantitative and/or qualitative research, expert opinion, and policy document).

*From:* Tricco AC, Lillie E, Zarin W, O'Brien KK, Colquhoun H, Levac D, et al. PRISMA Extension for Scoping Reviews (PRISMAScR): Checklist and Explanation. Ann Intern Med. 2018;169:467–473. [doi: 10.7326/M18-0850](http://annals.org/aim/fullarticle/2700389/prisma-extension-scoping-reviews-prisma-scr-checklist-explanation).
